# Correction: The critical role of novel benzophenone analogs on tumor growth inhibition targeting angiogenesis and apoptosis

**DOI:** 10.1039/c8md90018c

**Published:** 2018-04-10

**Authors:** Yasser Hussein Eissa Mohammed, Shaukath Ara Khanum

**Affiliations:** a Department of Chemistry , Yuvaraja's College , University of Mysore , Mysore -570005 , Karnataka , India . Email: shaukathara@yahoo.co.in ; Fax: +821 2419239 ; Tel: +91 99018 88755; b Department of Biochemistry , Faculty of Applied Science College , University of Hajjah , Yemen

## Abstract

Correction for ‘The critical role of novel benzophenone analogs on tumor growth inhibition targeting angiogenesis and apoptosis’ by Yasser Hussein Eissa Mohammed *et al.*, *Med. Chem. Commun.*, 2018, DOI: ; 10.1039/c7md00593h.



## 

The authors regret that there were some errors with the compound naming in their manuscript. The following are the corrected names for compounds **5b**, **5d**, **9a–d** and **10a–d**:

**5b**: [2-chloro-6-fluoro-4-(4-fluorobenzoyl)phenoxy]acetic acid ethyl ester.

**5d**: [2-chloro-6-fluoro-4-(4-methylbenzoyl)phenoxy]acetic acid ethyl ester.

**9a**: *N′*-(2-(4-benzoylphenoxy)acetyl)-1-methyl-1*H*-imidazole-4-carbohydrazide.

**9b**: *N′*-(2-(2-chloro-6-fluoro-4-(4-fluorobenzoyl)phenoxy)acetyl)-1-methyl-1*H*-imidazole-4-carbohydrazide.

**9c**: *N′*-(2-(2-chloro-4-(4-chlorobenzoyl)-6-fluorophenoxy)acetyl)-1-methyl-1*H*-imidazole-4-carbohydrazide.

**9d**: *N′*-(2-(2-chloro-6-fluoro-4-(4-methylbenzoyl)phenoxy)acetyl)-1-methyl-1*H*-imidazole-4-carbohydrazide.

**10a**: *N′*-(2-(4-benzoylphenoxy)acetyl)-2-oxo-2*H*-pyran-5-carbohydrazide.

**10b**: *N′*-(2-(2-chloro-6-fluoro-4-(4-fluorobenzoyl)phenoxy)acetyl)-2-oxo-2*H*-pyran-5-carbohydrazide.

**10c**: *N′*-(2-(2-chloro-4-(4-chlorobenzoyl)-6-fluorophenoxy)acetyl)-2-oxo-2*H*-pyran-5-carbohydrazide.

**10d**: *N′*-(2-(2-chloro-6-fluoro-4-(4-methylbenzoyl)phenoxy)acetyl)-2-oxo-2*H*-pyran-5-carbohydrazide.

In addition, the structure of **9a** was correct in the main manuscript, but wrong in the ESI. The ESI has been corrected.

The Royal Society of Chemistry apologises for these errors and any consequent inconvenience to authors and readers.

